# Greening Sport for Development and Peace: A Socio-Ecological Approach

**DOI:** 10.3389/fspor.2021.660743

**Published:** 2021-05-28

**Authors:** Richard Giulianotti

**Affiliations:** ^1^School of Sport, Exercise and Health Sciences, Loughborough University, Loughborough, United Kingdom; ^2^Department of Sports, Physical Education and Outdoor Studies, University of South-Eastern Norway, Kongsberg, Norway

**Keywords:** sport, development, peace, environment, socio-ecological approach

## Abstract

The global “sport for development and peace” (SDP) sector uses sport as a field of social activity to promote diverse types of non-sport social development. In this short perspective article, I critically examine and advocate the engagement of SDP with environmental issues. I argue for the adoption of a “socio-ecological” approach, to enable a greening of SDP that promotes both environmental and social justice. To that end, the article is organized into four main parts. First, I situate the discussion with respect to key literature on SDP and the environment. I then outline some of the main contextual factors that need to be considered on sport, development, and the environment. Third, I set out several core principles that should underpin the socio-ecological greening of SDP. Fourth, I examine how these principles may be implemented within SDP.

## Introduction

The global “sport for development and peace” (SDP) sector uses sport as a field of social activity to promote diverse types of non-sport social development. Most SDP work is undertaken by non-governmental organizations (NGOs) and, to a lesser extent, sport clubs or other agencies, through intervention programs with socially disadvantaged or “at-risk” young people. SDP program objectives usually align closely with the United Nations' development agenda, as encapsulated by the Millennium Development Goals (from 2000 to 2015) and the Sustainable Development Goals (SDGs, from 2015 to 2030) (see Collison et al., 2018).

The SDGs feature 17 different development goals, at least 12 of which are salient to the environment. These include commitments to

- sustainable management of water and sanitation [SDG 6];
- sustainable and modern energy [7];
- urgently combatting climate change [13];
- protecting the oceans, seas, and marine resources [14]; and
- and protecting terrestrial ecosystems [15].

         [United Nations (UN), 2015; Giulianotti et al., 2018].

In this short perspective article, I critically examine and advocate the engagement of SDP with environmental issues. I argue for the adoption of a “socio-ecological” approach, to enable a “greening” of SDP that promotes both environmental and social justice. To that end, the article is organized into four main parts. First, I situate the discussion with respect to key literature on SDP and the environment. I then outline some of the main contextual factors that need to be considered on sport, development, and the environment. Third, I set out several core principles that should underpin the socio-ecological greening of SDP. Fourth, I examine how these principles may be embedded within SDP.

## SDP and the Environment: Prior Literature

While there has been a relative lack of literature on SDP and the environment, growing interest in this area in recent years is noteworthy.^1^ In the context of this short article, two key prior publications may be considered, both of which are underpinned by critical sociological and anthropological standpoints.

First, the earliest full article on SDP and the environment, by Giulianotti et al. (2018), highlights the centrality of green issues to the SDGs and the accordant need for the SDP sector to attend fully to this challenge. Drawing on research across five international locations, Giulianotti et al. find that diverse SDP stakeholders, such as non-governmental and intergovernmental organizations, have largely overlooked environmental issues. In response, the authors note *inter alia* the pressing requirement to address the social inequalities of climate change. They also advance two concepts that may be utilized in future SDP/environment studies. First, the concept of “ecological modernization” draws critical scrutiny toward policy assumptions that environmental degradation will be successfully tackled through future scientific advances (see also Millington and Wilson, 2013). Second, the authors' concept of “benign governmentality” points to the ways in which positive civic conduct, such as everyday care for the environment, may be cultivated among young people through their participation within SDP programs and initiatives.

Second, the edited collection by Millington and Darnell (2019) features diverse studies of sport, development, and environmental issues by social scientists based mainly in North America, particularly Canada. While most articles concentrate on “development of sport” themes (e.g., sport mega events, sport and urban development, golf courses, and environmental politics), the contribution by Paraschak and Heine (2019) is particularly valuable here. These scholars examine how SDP and related development programs may engage with the “land-focused” activities and broader traditional physical cultures of indigenous peoples in Canada and elsewhere. Such an approach, they indicate, would have a variety of environmental, social, and cultural benefits, such as deepening community “ties to the land,” cultivating a “noncompetitive logic” in physical activity, culturally empowering indigenous peoples, and promoting a “long-term commitment to addressing all forms of environmental degradation” (Paraschak and Heine, 2019, p. 190–191).

With these contributions in mind—particularly, their calls for SDP to address environmental issues, social inequalities, community empowerment, and local and indigenous belief systems—I turn now to develop the case for the “greening” of SDP.

## Sport, Development, and the Environment: Broad Contextual Issues

To begin that task, we need to be *au fait* with the key issues and challenges that are faced by green SDP and global development agendas within the academic and policy fields. Three succinct points might be made here.

First, we appreciate that the SDGs and SDPs are not uncontested: many policymakers, academics, and publics hold deeply skeptical, even hostile, views on their value. Reflecting these diffuse, largely conservative approaches, the economist William Easterly (2015), writing in the influential periodical *Foreign Policy*, lampooned the SDGs as “senseless, dreamy, garbled” and mocked their message as: “Play sports! Be in harmony with nature! And end all preventable deaths! Only the U.N. could have come up with a document so worthless.” Easterly continued:

*[T]he SDGs are so encyclopedic that everything is top priority, which means nothing is a priority: “Sport is also an important enabler of sustainable development.” “Recognize and value… domestic work… and the promotion of shared responsibility within the household.” It's unclear how the U.N. is going to get more women to play soccer and more men to do the dishes*.

Such a standpoint on the SDGs, and on sport's role in promoting development, may be located within a spectrum of very diverse economic and policy approaches that are generally neoliberal or libertarian in nature. Such approaches tend to be intuitively hostile to environmentalism, multilateralism, and international institutions such as the UN and WHO and toward green or other social interventions that may restrict free-market agency or economic growth.

Second, from a broadly opposite position, an array of critical, radical, and progressive approaches highlight the need for deep power inequalities and pressing social justice issues to be central to a transformed global political–economic or development agenda. The more critical approaches posit, for example, that “sustainable development” is an oxymoron that carries inescapable, negative environmental impacts [Fletcher and Rammelt, 2017; Spaiser et al., 2017; cf. International Monetary Fund (IMF), 2020] and that the global development agenda is determined by the neoliberal, neocolonial, “WEIRD” interests of global capitalism (cf. Amin, 2006).^2^ For some anthropologists, the SDGs are the latest installment in postcolonial development, which evades political choices, marshals a technocratic “audit culture,” and redefines development failures as instructive successes (Ferguson, 1990; Scott, 1998; Strathern, 2000; Merry, 2016; Fukuda-Parr, 2017). Thus, for other analysts, development should focus less on SDG targets and more on “actual social struggles” concerning poverty and democracy (Bond, 2006).

Third, I recognize some diverse, positive signs and trends in global development, including on the environment. These indicate some shifts away from policies that equate development with free markets and economic growth. Consider, for example, state economic interventions, backed by global institutions (IMF, World Bank, UN), during the COVID-19 (coronavirus disease 2019) pandemic, or even the claims from global business (albeit yet to be adequately tested) that “shareholder capitalism” is over and that “stakeholder capitalism” is the way forward, including recognition of the need to tackle climate change and to protect the environment.^3^ In addition, the SDGs encapsulate how global institutions place a stronger premium on the non-economic, social, and environmental aspects of development (Macdonald and Ruckert, 2009; Peck et al., 2010; Elwood et al., 2017; Raworth, 2017; Hickel, 2020). Indicatively, the UN's *Human Development Report 2020*, entitled *The Next Frontier: Human Development and the Anthropocene*, focuses squarely on combatting ecological devastation through different forms of environmental justice. It includes calls to empower communities, deliver better education, “learn from locals,” and engage with “indigenous and local knowledge systems and practices” (UN, 2020).

The “socio-ecological” approach that I advocate for SDP and wider development must be located within these three broad policy contexts. This approach is aligned with the critical, progressive standpoints on development outlined here and seeks to build on the positive signs and trends in this direction in recent times.

## Greening SDP: A Socio-ecological Approach

The socio-ecological approach to development combines environmental and social justice. It is “attentive to ways in which social and ecological systems are intertwined in ways that are currently driving ecological devastation and social inequality but which might be transformed along more sustainable and socially just lines” (Newell, 2020, p. 5). To pursue the greening of SDP, I argue that the socio-ecological approach should have four main pillars, relating to environmental protection, social equality, democracy, and social justice.

First, any development philosophy or strategy must be anchored in a fundamental commitment to genuinely sustainable development, which protects, preserves, and nurtures the natural environment. In line with earlier work (Millington and Wilson, 2013; Giulianotti et al., 2018), I posit that such development must eschew convenient, Micawberish modernization theories, which assume that future modern science will “turn up something” to reverse the ongoing environmental devastation of our planet.

Second, a socio-ecological approach tackles environmental and social inequalities and injustices (cf. Cole and Foster, 2001). The interdependencies of the environmental and the social are clear. As Chancel (2020) reminds us, the global poor are the most threatened by climate and other environmental changes (e.g., urban pollution, water shortages, land desertification, extreme weather), and they are the least responsible for these environmental changes. Yet, they are the most in need of economic development, to rise out of poverty, and they are the most threatened by green policies that cut jobs or growth. Thus, we must address SDGs that focus on the environment *and* on inequality; the latter include SDG10 (reduce inequality) and SDG5 (gender equality).

Social and environmental inequalities reflect systemic differences in wealth and power at local, national, regional, and transnational levels. Crucially, they also operate through deep “recognition gaps,” which Lamont defines as “disparities in worth and cultural membership between groups in a society” (2018, p. 421–2). These social divisions shape the diverging, often opposing interests, between groups over social justice and environmental protection.

In the global development context, greatest focus is understandably placed on transnational inequalities, along global North/South lines. Yet, there are also deeply entrenched inequalities *within* low- and middle-income countries to consider. In the Indian megacity of Delhi, Baviskar (2011, 2021) argues that *bourgeois environmentalism*—that is, the environmental and socioeconomic interests of the middle and upper classes—directly threatens the shelter, livelihoods, and communities of millions of urban poor. Baviskar posits that bourgeois environmentalism features “the (mainly) middle-class pursuit of order, hygiene and safety, and ecological conservation”; “mobilize[s] the discourse of “public interest”; and “citizenship” to advance elite interests through the media, courts, and state; protects “luxury emissions” and “resource-intensive affluent lifestyles” (e.g., cars, consumerism); and harbors a deep “hostility to the poor in the pursuit of a ‘clean and green' environment, where the very presence of the poor is equated with pollution” (Baviskar, 2011, 2021, p. 110, 159). Confronting their hypocrisy, Baviskar (2021, p. 217) asks:

*Where are bourgeois environmentalists when air pollution peaks? What do they do when Delhi is hit by a heatwave? Well, they travel in air-conditioned cars from air-conditioned homes to air-conditioned offices, restaurants, and shops. They compare brands of masks and air purifiers. They complain on social media about farmers burning stubble in Punjab and how the ban on firecrackers is being flouted. When it gets too much, they retreat to the hills in time-honored colonial fashion. Meanwhile, the exhaust from their vehicles and appliances makes the air hotter and dirtier for everyone else*.

Thus, a socio-ecological approach must attend to the deep social divisions of wealth, power, and recognition that operate along local and national as well as transnational lines.

Third, the socio-ecological approach needs to be founded on intensified, multi-level, fully inclusive democratization. At grassroots level, an adapted version of the idea of “associative democracy,” as initially advanced by Hirst (1993), should be pursued. The definitive principle of associative democracy is that “the freedom of individuals is best enabled by association—by working and engaging with others on a democratic and voluntary (freely entered into) basis” (Westall, 2011, p. 8). In the SDP or wider development context, associative democracy nurtures empowering, resource-supported, community-level frameworks of decision-making that are conducive to locally impactful environment-related work. It repudiates models of hierarchical, vertical, or authoritarian developmentalism. It affords instead a potentially robust sociopolitical framework for facilitating community voice and decision-making on SDP, the environment, and development strategies. Thus, such democratization must privilege local communities, especially the most marginalized, in agenda-setting and decision-making. Additionally, it must encompass the full spectrum of organizational stakeholders, from established national and international governmental agencies, corporations and businesses, NGOs, and social enterprises, through to more “disruptive” campaign groups, community-based organizations, local associations, and social movements (Kaldor, 2005; Giulianotti, 2011).

Finally, a socio-ecological approach should be underpinned by an integration of the two standpoints on justice—the transcendental and the comparative—that are outlined by Sen (2009). The idealist, transcendental standpoint pictures how an unblemished, perfectly just society should look; the more practical, comparative standpoint lists and compares different types or instances of (in)justice (cf. Case and Deaton, 2020, p. 245). While Sen (2009) advocates the latter, comparative approach, I would argue that, at least in the case of green SDP and wider development activity, the two standpoints should be combined. Thus, an idealist standpoint is needed to inspire and to mobilize different stakeholders with a vision of environmental sustainability that they can work toward; meanwhile, the practical standpoint is needed to enable these stakeholders to prioritize issues and problems and to organize and to direct their activities in pragmatic, progressive fashion.

## Implementing a Socio-ecological Approach in SDP

Based on these four pillars, I outline and illustrate seven ways in which this socio-ecological approach may be implemented to advance the greening of SDP.

### Mainstreaming the Environment

SDP organizational stakeholders should position the environment consistently within their policies, programs, partnerships, and practices. This would replicate attempts to mainstream other critical development themes—such as on gender and disability—within SDP. There are many ways to do so. For example, all SDP programs should include some environmental components, such as community “cleanups,” or educational activities on local environmental hazards and the impacts of climate change (see MYSA, 1992). SDP social enterprises may pursue ecofriendly commercial activities such as plastic recycling businesses that employ or support marginalized young people. More broadly, in establishing partnerships, NGOs and other organizations should ensure that at least some of their collective focus is on tackling critical community-level issues relating to climate change and environmental protection.

### Democratization

Socio-ecological approaches in SDP should draw on the inclusive principles of associative democracy, devolving decision-making to community levels and fully engaging marginalized local voices. For example, the planning and implementation of SDP programs might be led by a forum of local people, including strong representation of women, young people, and lower-class/caste community members. This forum would be especially valuable in identifying key socio-ecological needs and issues within the community.

### Social Inequalities—SDP as a “Recognition Space”

Socio-ecological SDP must be not only a “safe space” but also a “recognition space” for the full, active participation of the most marginalized social groups. Thus, for example, NGO officials, volunteers, and program participants should be drawn from different segments of the local community, but especially from lower-status groups. Lower-status officials and volunteers may be given prominent leadership and decision-making roles. Lower-status community members should be given particular recognition and voice within community forums in program planning. The NGO aims, objectives, and program activities should include strong commitments to, and educational messages on, the full recognition and equality of lower-status groups, especially in societies with very high levels of inequality.

### Combining Comparative and Transcendental Approaches

Socio-ecological SDP should combine both approaches to justice, in ways that prioritize the needs and interests of the most disadvantaged social groups. In comparative, practical terms, these activities might include, for example, environmental education programs, local cleanup and recycling activities, and public campaigns that pursue particular outcomes (such as in lobbying municipalities for changes in local traffic systems). In transcendental terms, the NGO should work with local people to develop a socio-ecological vision of the community that may be worked toward, which might include, for example, new housing, sanitation, and transport systems, or transformed public spaces for sport, physical activity, and recreation.

### Greening Programming

A socio-ecological approach is marked by flexible and holistic ways of planning, implementing, and assessing [i.e., monitoring, evaluating, learning (MEL)] programs, according to context. The specific socio-ecological conditions and needs of local people need to be carefully identified, to shape programs; for example, some communities may be badly affected by climate change and, for example, need better access to scarce water, or protection from flooding; others may be worse affected by waste and pollution and so may target cleanup initiatives, recycling schemes, or campaigns against polluting industries. NGOs should adapt delivery techniques to context, for example, in selecting sports or physical activities that will appeal to local young people. MEL should avoid “audit culture” approaches and instead utilize diverse, locally attuned methods and criteria of success. For example, in communities with strong narrative traditions, “storytelling” methods for data gathering will be valuable. MEL should also ensure that local knowledge systems and cultural beliefs on the environment are embedded in program implementation (e.g., through education sessions) and evaluation.

### The Sustainability of SDP and Sport

A socio-ecological approach requires all stakeholders to exercise critical reflexivity on the environmental sustainability of SDP and sport more broadly. Thus, for example, NGOs need to assess the environmental impacts or costs of key SDP activities, such as running intervention programs, the transportation of staff, volunteers and user groups to events and meetings, and the production of sport equipment or single-use materials utilized in programs.

### The Positions and Roles of Social Researchers

Here, I follow Lamont (2018) in arguing that academics, alongside other “key social actors” (such as “knowledge workers,” “cultural intermediaries,” and “social movement actors and leaders”), can play vital roles in pursuing social (and socio-ecological) change. In SDP, for example, academics may be advisory partners or “critical friends” for environment-related programs and campaigns, such as in assisting with organization, delivery, and MEL. More broadly, academic partners may also work with other stakeholders to ensure that socio-ecological principles and practices are integral to SDP programs.

## Concluding Comments

I have sought here to set out the case for a particular socio-ecological approach to policy and practice within SDP. This approach requires us to recognize the interdependence of environmental and social forms of (in)justice with respect to development; thus, we need to engage with SDGs that center on the environment, social inequality, and social exclusion. In preparing the ground for this approach, we need to be fully aware of critical contextual issues, challenges, and opportunities, including diverse skepticisms toward SDP and global development agendas. As highlighted, the socio-ecological approach has several strands and implementational aspects. These include, among other points, the needs to establish SDP as a “recognition space” that privileges marginalized groups, their social struggles, and environmental interests; to nurture “associative democracy” within local development contexts; and to pursue pragmatic and visionary approaches when establishing development aims, objectives, and priorities.

For academics in SDP, the socio-ecological approach offers rich opportunities for critical, participatory research that is socially and ecologically engaged. A final observation here concerns the need to pay attention to global scale. For social and environmental injustices to be tackled with maximum effect, actions and changes are needed at transnational levels. Similarly, for academics, if a green or socio-ecological approach is to have real consequence—whether in the academy or in the SDP and development field—then research dialogue and collaborations also need to proceed across a transnational terrain.

## Data Availability Statement

The original contributions presented in the study are included in the article/supplementary material, further inquiries can be directed to the corresponding author/s.

## Author Contributions

The author confirms being the sole contributor of this work and has approved it for publication.

## Conflict of Interest

The author declares that the research was conducted in the absence of any commercial or financial relationships that could be construed as a potential conflict of interest.

## Footnotes

^1^See, for example, Darnell's (2018) overview.

^2^Collier (2018, p. 12) introduced the acronym WEIRD, to refer to “Western, Educated, Industrial, Rich and Developed.”

^3^See https://opportunity.businessroundtable.org/ourcommitment/; https://www.weforum.org/agenda/2019/12/davos-manifesto-2020-the-universal-purpose-of-a-company-in-the-fourth-industrial-revolution/
